# Full Mouth Rehabilitation of an Ectodermal Dysplasia Patient with Implant-Supported Prostheses: A Clinical Report

**Published:** 2013-05

**Authors:** Rahab Ghoveizi, Hakimeh Siadat, Sakineh Nikzad, Ghasem Ommati Shabestari, Yadolah Soleimani Shayesteh

**Affiliations:** 1 Assistant Professor, Department of Prosthodontics, School of Dentistry, Shahid Beheshti University of Medical Sciences, Tehran, Iran; 2 Dental Implant Research Center, Associated Professor, Department of Prosthodontics, School of Dentistry, Tehran University of Medical Sciences, Tehran, Iran; 3 Associate Professor, Department of Prosthodontics, School of Dentistry, Tehran University of Medical Sciences, Tehran, Iran; 4 Associated Professor, Department of Prosthodontics, School of Dentistry, Tehran University of Medical Sciences, Tehran, Iran; 5 Professor, Department of Periodontics, School of Dentistry,Tehran University of Medical Sciences, Tehran, Iran

**Keywords:** Ectodermal Dysplasia; Dental Implants; Fixed Partial Dentures; Rehabilitation

## Abstract

Full mouth rehabilitation in patients with ectodermal dysplasia (ED) is difficult to manage, especially because the afflicted individuals are quite young when they are evaluated for treatment; therefore, esthetics is an important concern. This clinical report describes the rehabilitation of a 19-year-old girl diagnosed with ectodermal dysplasia. Eleven implants were placed in the maxilla and mandible along with bone grafting to the upper jaw and both arches were constructed by metal-ceramic implant-supported fixed prostheses. This treatment plan seems to be favorable for ED patients.

## Introduction

Ectodermal dysplasia (ED) is described as an inherited disorder characterized by the abnormality of at least two structures of ectodermal origin [1-3]. The different forms of ED lead to dysplasia or aplasia of ectodermal tissues, such as skin, hair, nails, sweat glands and the teeth[4]. The condition can be classified in two broad types: (1) hidrotic is an autosomal triad with normal sweat glands; and (2) anhidrotic (hypohidrotic) is an X-linked recessive triad with the absence of, or considerable decrease in the sweat glands [5-7]. 

Because X-linked is the most prevalent form, ED significantly involves more males and furthermore, the affected men often have extensive dental anomalies, such as missing most of their deciduous and permanent teeth [8-10].

The most common oral manifestations are hypodontia, anodontia, tapered or malformed teeth, delayed dental eruption and because there is a deficiency in the alveolar process, it often leads to the loss of vertical dimension [11-15].

External characteristics are an indistinct vermilion border, protuberant lips, a prominent forehead and a depressed nasal bridge [14,16].

**Fig 1:**
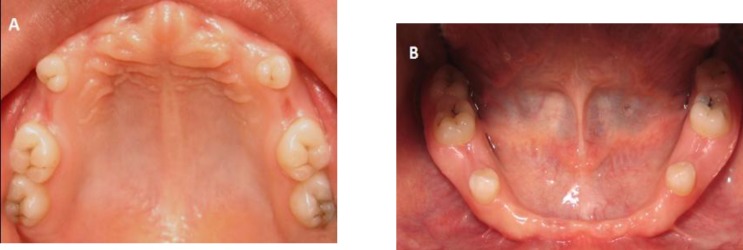
Pretreatment intraoral condition A. Intraoral view of the maxilla B. Intraoral view of the mandible

Placement of osseo-integrated implants when patients are in their late teens is well documented in literatures [1,2,3,7]. 

The main problem of this treatment is insufficient bone, particularly in the maxilla, so in some cases, bone grafting might be necessary [ 2,17]. 

Finally, a treatment plan should be personalized for every patient with ED and it depends on a significant number of factors, such as the patient's age, medical history, the number of present teeth, alveolar process, esthetics and the patient's attitudes.

The present study describes using osseointegrated dental implants to full mouth rehabilitate an ED patient with maxillary and mandibular oligodontia, and severe atrophy of the residual alveolar crest.

**CLINICAL REPORT**

A 19-year-old girl was referred to the Prosthodontic Department of Tehran University of Medical Sciences, with the chief complaint of unesthetic appearance and inefficient mastication. She was the only member of the family who suffered from ED.

The patient’s medical history showed that she had dyshidrosis (abnormal sweat glands).

There were no abnormal findings or contraindications to dental treatment.

Extraoral examination revealed frontal prominence, mild angle class III jaw relation and prominent lips. 

The intraoral examination in each quadrant of both maxillary and mandibular arches showed, that only the first permanent premolar and the first and second permanent molars were present and the other teeth were missing (Fig 1). All present teeth had small and deciduous-like crowns and there were also mild carries in all occlusal grooves of the molars. 

**Fig 2:**
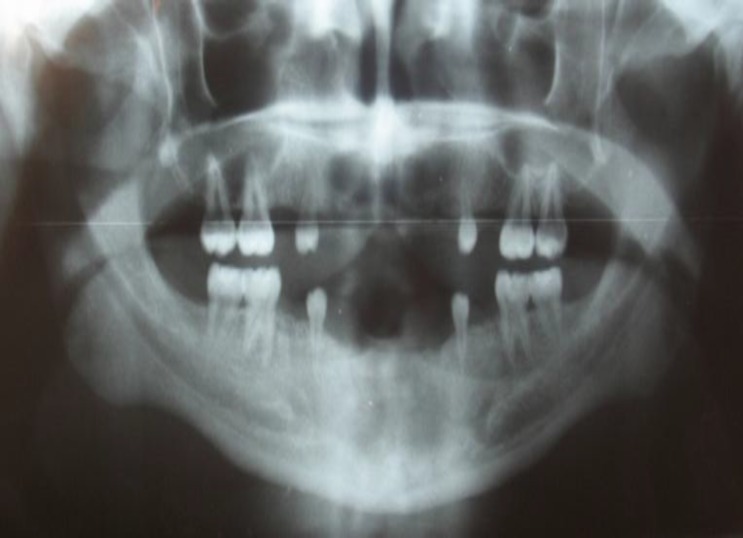
Preoperative panoramic radiograph

The patient exhibited loss of vertical dimension of occlusion (VDO) with under-developed alveolar ridges. Panoramic radiographic examination showed enlarged pulp chambers in all permanent molars and narrow and short roots in all first premolars (Fig 2). 

The lateral cephalographs and hand radiography demonstrated that her growth was complete. On the basis of clinical and radiographic examinations, the definitive treatment plan included fabrication of a maxillary and a mandibular implant-supported fixed prosthesis (FP2 according to Misch) and fixed partial denture (FPD) [18]. First, preliminary impressions were made with irreversible hydrocolloid (Alginat Super; Pluradent, Offenbach, Germany) and to make an interocclusal record, wax occlusal rims were formed on the acrylic resin record base to transmit the interarch relation to the articulator. 

**Fig3:**
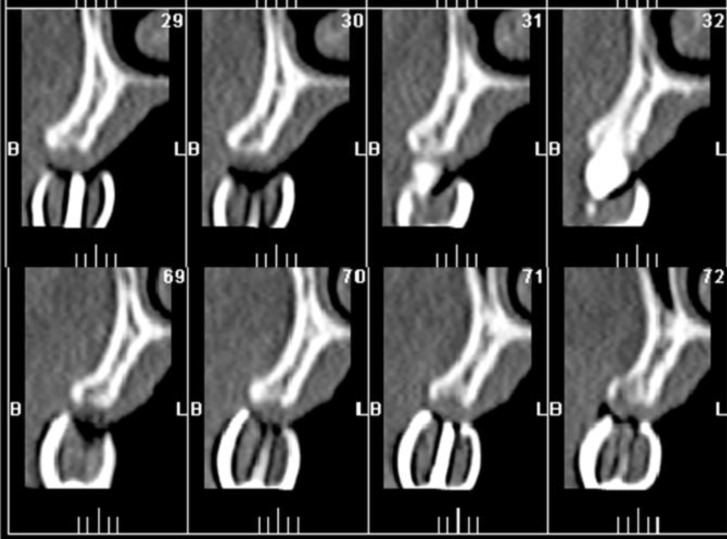
CT evaluation with radiographic stent

**Fig4:**
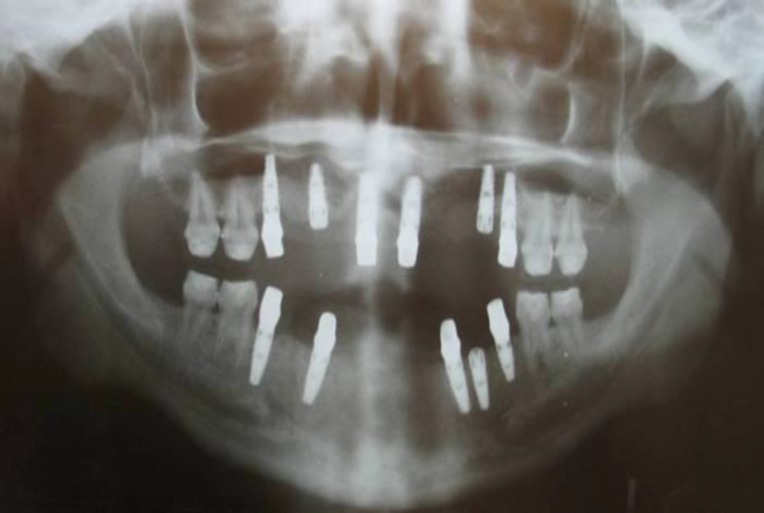
Postoperative panoramic radiograph of osseointegrated implants

After completing the trial arrangement of the teeth (Physiodens; Vita Zahnfabrik, Bad Sackingen, Germany), they were positioned intraorally to evaluate the esthetics, teeth positions and proper occlusal relations. For computerized tomographic scan (CT scan) purpose, the trial denture was converted to a radiographic guide. The acrylic teeth were coated with barium sulfate and gutta percha was inserted into the long axes of every teeth. This made the teeth opaque so that the occlusal plane and the long axes of every tooth could be detected in CT scan (Fig 3). The data acquired from radiographic survey were utilized to determine implant positions and fabrication of the surgical guide. Because of the narrow and short roots of all the second premolars, they were extracted six months before implant surgery. Eleven implants were planned in both the maxillary and mandibular arches. Due to the lack of sufficient bone width, narrow implants were chosen and bone augmentation was done simultaneously. 

Five 3.5×10.5mm and one 3.5×8mm TBR implants (Connect system, Toulouse, France) were placed in the maxilla, and four 3.5×10.5mm and one 3.5×8mm TBR implants were placed in the mandible (Fig 4). 

After a six-month healing period, due to the small size of the present teeth, all the first and second molars were prepared with the minimal occlusal and axial reduction (0.5mm and 0.9mm respectively) with a moderate chamfer margin. Preliminary impressions were made using irreversible hydrocolloid impression material (Alginat Super; Pluradent, Offenbach). Custom open trays (SR Ivolen, Ivoclar, Vivadent, Schaan, Liechtenstein) were fabricated on these casts for final impressions. Screw-retained impression copings (O-TD300, TBR) were fastened to the implants, and final impressions of the implants and prepared teeth were made with vinyl polysiloxane impression material (Affinis, Colt`ene AG, Feldwiesenstrasse Altst¨atten, Switzerland) using an open-tray technique [19]. Each impression was poured with type IV dental stone (Resin Rock; Whip Mix Corp, Louisville, Ky). 

**Fig5:**
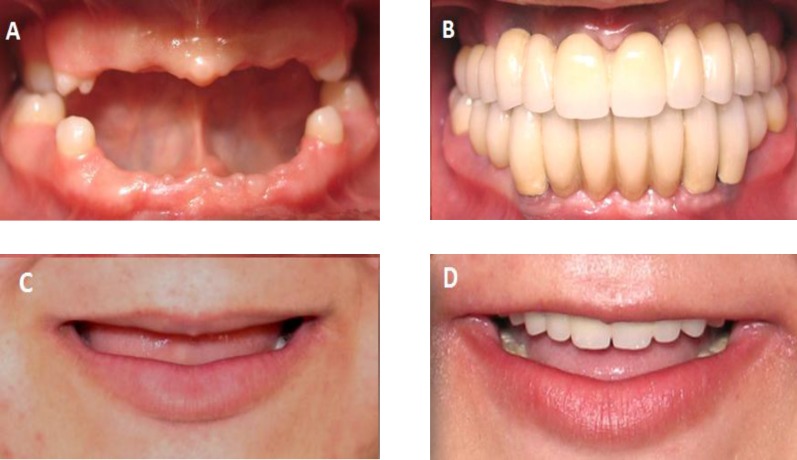
A. C. Patients' occlusion before treatment B. Definitive cement-retained restorations in place C, Patients' smile design before treatment D, Patients' smile design after treatment

To obtain accurate centric relation records, screw-retained record bases were fabricated on the master casts [19]. Casts were mounted on a semi adjustable articulator (Denar Mark II, Teledyne Water Pik, Fort Collins, CO) using centric relation and an arbitrary facebow record (Denar Slidematic, Teledyne Water Pik).The first step to fabricate the definitive prostheses depended on ascertaining the position of the artificial teeth in relation to the definitive casts. As a result, to transfer interarch relationship and to check esthetics and phonetics intraorally, wax trial dentures were fabricated on the definitive casts.

Following this, temporary abutments were selected and acrylic resin fixed provisional prostheses were constructed on the definitive casts. A mutually protected occlusion was verified in the screw-retained acrylic resin fixed provisional prostheses intraorally.

The interim fixed restorations were utilied to evaluate definitive prosthesis positions after 12 weeks of comfortable function. 

A custom mold was made with polyvinyl siloxane impression material (Rapid; Coltene AG, Altstatten, Switzerland) on the patient’s fixed provisional prostheses to reproduce the dimensions. This index was used as a guide in the abutment selection and full-contour waxing of the definitive prostheses. After abutment selection, framework patterns were casted in a base metal alloy (Palladium-Silver Alloy; Ivoclar Vivadent, Schaan, Liechtenstein). The fitness of the metal frameworks were clinically and radiographically evaluated in the mouth. The veneering porcelain (Nobel Rondo; Nobel Biocare AB) was applied on the frameworks and fired according to the manufacturer’s instructions. The metal ceramic restorations were evaluated to develop a mutually protected occlusion. Then restorations were characterized and finally glazed. At delivery, all abutment screws were torqued to 30 N_cm as suggested by the manufacturer. 

The screw access channel was filled by putty (Lab-Putty; Coltene/Whaledent, Cuyahoga Falls) and final restorations were cemented using a resin modified glass ionomer (FujiCEM; GC America, Alsip, Ill) (Fig 5). Postoperative instructions were given to the patient and a panoramic radiograph was taken. The patient has been followed for 2 years without any complication and to date the results have been satisfactory.

## Discussion

Oral rehabilitation of ED patients requires teamwork endeavors, and treatment of ED is a controversial issue. Osseo-integrated implants often provide more retention and esthetics for young adult patients. Because some patients are still in their growing period furthermore, due to the lack of sufficient bone in the jaws, dental implants should be used carefully [20].

In contrast, according to Celar et al [21], since implant placement can improve denture stability, esthetics and function, growing patients can benefit from it. In addition, Escobar and Epker [22]. advocate that implant insertion in ED children can stimulate alveolar development. As a consequence, to achieve the best result, the treatment plan should be individualized for every patient with ED.
